# The Relationship between Personality Traits with Depressive Symptoms and Suicidal Ideation among Medical Students: A Cross-Sectional Study at One Medical School in Germany

**DOI:** 10.3390/ijerph15071462

**Published:** 2018-07-11

**Authors:** Winnie S Chow, Jan Schmidtke, Adrian Loerbroks, Thomas Muth, Peter Angerer

**Affiliations:** Institute of Occupational, Social and Environmental Medicine, Centre for Health and Society, Faculty of Medicine, University of Düsseldorf, 40225 Düsseldorf, Germany; Jan.Schmidtke@uni-duesseldorf.de (J.S.); Adrian.Loerbroks@uni-duesseldorf.de (A.L.); Thomas.Muth@hhu.de (T.M.); peter.angerer@uni-duesseldorf.de (P.A.)

**Keywords:** mental health, medical students, academic stress, personality traits, depressive symptoms, suicidal ideation

## Abstract

Medical students are at increased risk of experiencing mental health problems. Certain personality traits may be associated with elevated vulnerability to study-related stress and poor mental health. This study aimed to investigate the relationship between such personality traits and mental health outcomes among medical students. We drew on cross-sectional data from 251 medical students who had been enrolled for one-year at a medical school in Germany. Depressive symptoms were measured using the Patient Health Questionnaire-8 (PHQ-8) and suicidal ideation was assessed by item 9 from the Patient Health Questionnaire-9 (PHQ-9). Personality traits were captured using the Business-Focused Inventory of Personality 6 Factors (BIP-6F). Multivariable logistic regression analyses were used to quantify the associations between work-related personality factors and mental health outcomes, controlling for demographic and social factors. Odds ratios (ORs) as outcome measures with 95% confidence intervals (CIs) were used. After controlling for important confounders, medical students who scored highly on Stability had lower odds of depressive symptoms (OR: 0.19, 95% CI: 0.09–0.42, *p* < 0.001) and suicidality (OR: 0.38, 95% CI: 0.16–0.87, *p* < 0.05) than those with high scores in other work-related personality factors. Findings also showed that those who scored highly on Dominance had greater odds of depressive symptoms (OR: 2.46, 95% CI: 1.22–4.97), *p* < 0.01). Work-related personality-informed interventions, which promote students’ mental well-being and reduce academic stress should be considered at various stages of their medical training.

## 1. Introduction

Academic stress among medical students has repeatedly been linked to poorer health outcomes, such as a reduced quality of life [1,2], musculoskeletal complaints [3,4], and depressive and anxiety symptoms [5,6,7]. Depression and suicidal ideation are major public health concerns in medical schools. For instance, Givens and Tjia [8] found that about one quarter of first and second year medical students showed signs of depression. Another survey carried out by Student BMJ [9] in the UK showed that approximately 30% of medical students had a mental illness or had received treatment. Evidence presented in a study from Germany indicates that newly enrolled medical students have a higher prevalence of psychosomatic complaints and panic disorders compared to the general population [5]. Furthermore, high prevalence of suicidal ideation among medical students has been established globally [9,10,11,12,13,14]. Other major risk factors for suicidality include hopelessness and sleep-related disorders [15]. Due to heavy academic workload and high levels of stress in medical training, medical students are particularly vulnerable to poor sleep or sleep related problems [16].

Personality traits have been shown to be good predictors of academic success during medical training [17]. Yet, previous studies have suggested that some specific personality predispositions based on the five-factor model may be more vulnerable to stress [18,19] and poorer mental health [20,21]. It has been put forward that medical students with more pronounced levels of neuroticism (a personality dimension measured on a continuum, ranging from emotional stability (low neuroticism) to emotional instability (high neuroticism)) are at increased risk for depression [21] and suicidal ideation [22]. Neuroticism was found to be negatively linked to the severity of depressive symptoms [20] and anxiety symptoms, while positively associated to stress vulnerability [19]. Among temperament traits, some specific affective temperaments have also been shown to increase the risk of suicide [23,24]. Pompili et al. [24], for example, reported that cyclothymic, irritable, anxious, depressive affective temperaments have a strong relationship with suicidal behavior.

Taken together, specific Big-Five personality traits appear to be linked to poorer mental health and an elevated susceptibility to experiencing stress. However, to our knowledge, no studies have been done on the relationship between work-related personality traits and mental health in medical students. As described above, most research examining the relationship between personality traits and poor mental health among medical students has utilized instruments assessing the Big-Five personality dimensions, reflecting personality traits in non-work-related contexts [18,19,20,21,22]. However, less attention has been paid to work-related personality traits, namely behaviors and personality traits in a professional context (e.g., at work). Work-related personality traits focus solely on activities in a professional context. For example, the Commitment personality factor is linked to behaviors and situations at work and is referring only to a person’s level of commitment at pursuing his or her professional goals. Similarly, the Stability personality factor merely explores how well a person manages and copes under pressure at work. All medical students must undergo personal and professional development, extensive practical training hours and must carry out basic clinical work as part of their medical training. In order to have low medical school attrition rate [25], and to reduce the high number of young doctors with poor mental health after graduation from medical school [26], it is necessary to detect individuals who are at risk of developing mental health problems early and to identify prevention strategies and appropriate treatment. To address these concerns, the present study extends previous research by estimating the prevalence of depressive symptoms and suicidal ideation as well as exploring the relationship between work-related personality traits and depressive symptoms and suicidal ideation in a sample of medical students after one-year enrollment. Academic stress at medical schools has been found to be severe and mental health has been shown to worsen after students begin medical school, as it is a difficult time of transition from adolescence into early adulthood [27]. Identifying the underlying determinants of poor mental health and susceptibility to academic/psychosocial stress among medical students is vital for health monitoring, wellness/promotion preventive wellness services, stress management and later the junior doctors’ training period.

Therefore, this study addressed the following questions. What is the prevalence of depression and suicidal ideation among medical students after one-year enrollment of their medical training, namely at the beginning of the third semester? Is there an association of work-related personality traits with depressive symptomology and suicidal ideation? Consistent with the previous findings presented above, it can be assumed that individuals with low neuroticism personality trait scores have poorer mental health. Hence, we postulated that medical students with higher scores on the Stability work-based personality trait would be associated with lower odds of depressive symptoms and suicidality than those with high scores in other work-based personality traits.

## 2. Materials and Methods

### 2.1. Study Design and Participants

This was a cross-sectional study of students enrolled at the medical school of the University of Düsseldorf, Germany. The medical students were participants of an ongoing prospective cohort study (i.e., “Healthy Learning in Düsseldorf study” (HELD)). We focused on students enrolled in a newly structured curriculum (“Modellstudiengang”). They started their medical training in October 2013 (Cohort 2) and October 2016 (Cohort 3). Students were invited to take part in the study at the beginning of their third semester. Data were collected in October–December 2014 (Cohort 2) and 2017 (Cohort 3).

Eight hundred five medical students were invited to participate in the study. A total of 437 medical students (response rate 53.4%) completed the questionnaire. This study focused on all participants that had no missing data among all variables of interest. Thus, this present investigation focused on the 251 medical students who participated after one-year of enrollment, namely at the start of the third semester. Given that the major characteristics of the medical students from the two cohorts were similar and analyses further showed that both cohorts were comparable; these data were pooled together. This study was approved by the Ethics Committee of Heinrich Heine University. Participation in this study was voluntary and written informed consent was obtained from each individual.

### 2.2. Measures

Work-related personality dimensions were measured by the Business-focused Inventory of Personality 6 Factors (BIP-6F), whose psychometric properties have been documented [28]. BIP-6F covers six major dimensions (Commitment, Discipline, Social Competence, Cooperation, Dominance and Stability) by 48 items. Within each factor, so-called facets (e.g., career orientation, or tolerance to frustration) can be identified on a lower level of abstraction that describes specific personality dimensions (e.g., Commitment or Stability), respectively. Respondents specify their level of agreement with each statement on a six point Likert rating scale. In other words, the students were asked to assess their own behavior in a professional context by rating how well each of the 48 statements applied to them in a work environment, specifically their medical training. Table 1 shows the main focus of each of the six factors, as well as which facets relate to which factors [28].

Each factor corresponds to six items. Mean scores were then calculated across the items constituting a given dimension, to obtain scores ranging from 1 to 6, with 6 indicating a high accordance with the respective factor. For statistical analyses, factor-specific BIP-6F scores were dichotomized at the median, coded as 0 (low; the reference group) if below, as 1 (high) if above the median.

Depressive symptoms were measured using the 8-item Patient Health Questionnaire (PHQ-8). This scale has been validated and used in a range of different populations and settings [29]. Individuals were asked to rate how often, over the past two weeks, they experienced each of the depressive symptoms, with the following response options: 0 = not at all, 1 = several days, 2 = more than half the days, and 3 = nearly every day. A cut-off score of 10 has been identified as discriminating major depression [30] and was accordingly used in our statistical analysis.

Suicidal ideation was measured by a single item of the PHQ-9 (i.e., item #9). The assessment of suicidal ideation based on such a single item is widely used in epidemiology studies to indicate the presence of suicidal ideation [31]. Participants were asked about suicidal thoughts and behaviors (“thoughts that you would be better off dead or of hurting yourself in some way”). Suicidal thoughts were then coded as 1 = ’Yes’ if the respondent mentioned at least ‘on several days’, 0 = ‘No’ if mentioned ‘not at all’.

#### Confounders

Previous research has demonstrated that age, gender, being in a partnership, and (parental) socioeconomic status are important determinants of mental health [32,33,34,35,36]. We adjusted for those confounders in the current study. Age was indicated in years. Gender was coded either 1 (female) or 0 (male). The groups coded 0s were the reference group for gender.

Participants were asked if they have a steady partner. Responses were coded as 0 if ‘No’ and 1 if ’Yes’. In terms of socioeconomic status, participants were then asked how they would describe the financial situation in their parental home?”, using a scale ranging from 1 = very poor to 10 = very rich. bad. 

### 2.3. Statistical Analysis

First, descriptive statistics were used to explore sample characteristics, mainly to show the six occupational/job related personality dimensions (Commitment, Discipline, Social Competence, Cooperation, Dominance and Stability) and the prevalence of depressive symptoms and suicidal ideation. Following this, multivariable logistic regression analyses were carried out to explore the association between work-related personality traits with two mental health indicators (outcome variable). The first models assessed the association without controlling for covariates. In the second models, demographic factors were adjusted. Finally, social-economic factors were adjusted in the third models. Separate regression models were calculated for each mental health indicator and adjusted for the covariates mentioned above, one by one. All estimates were reported as odds ratios (OR) and corresponding 95% confidence intervals (CIs). To assess goodness of fit of the models, pseudo-R^2^ (Nagelkerke) was also calculated. Sensitivity analyses were also performed to examine the robustness of the associations. First, we compared results of main analyses (BIP-6F dichotomized at the medians) to results of the sensitivity analyses (BIP-6F dichotomized at the upper tertile). To further increase confidence in our findings, we also compared results of main analyses (depression symptoms using PHQ-8) to results of the sensitivity analyses (depression symptoms using PHQ-9). The IBM SPSS version 24 was used for the analyses.

## 3. Results

A total of 437 medical students filled out the self-reported questionnaire. One-hundred-eighty-six medical students were excluded due to absent information or incomplete data for main studied variables. The final sample included in the analysis was 251. Table 2 shows the characteristics of the study population. The average age of the medical students was 22 years and more than half of the participants were female (71.3%). Symptoms of depression and suicidal ideation showed high prevalence among medical students after one-year enrollment (at the start of their third semester) equaling 20.7% and 14.7%, respectively (Table 1).

With respect to our main research question, the Stability work-related personality factor was significantly associated with depression and suicidal ideation. Social Competence was significantly associated with depression. However, it remains unclear whether the association can be attributed to other factors such as demographic and social factors. To control for this problem, we calculated multiple logistic regression models and adjusted for important confounders. The results of these analyses are presented in the Table 3, where models 2 and 3 indicate the adjusted odds ratios and 95% confidence interval.

Table 3 presents the associations between work-related personality factors and the two mental health outcomes, separately for depressive symptoms and suicidal ideation. All three models were overall stable regardless of adjustment. Controlling for the different confounders did not substantially change the estimates of the association.

In model 3, adjusting for socio factors (relationship and socio-economic status), both Stability and Dominance remained significant for depression. High scores on Stability remained as a protective factor against depression (OR: 0.19, 95% CI: 0.09–0.42, *p* < 0.001) and suicidal ideation (OR: 0.37, 95% CI: 0.16–0.87, *p* < 0.01). Sensitivity analyses were performed to assess the robustness of the primary results for depression symptoms using PHQ-8. Both primary (PHQ-8) and sensitivity analyses (PHQ-9) showed no marked differences between these two validated scales for depression symptoms in our study.

## 4. Discussion

To our knowledge, this is the first study examining the association between work-related personality factors with depressive symptoms and suicidal ideation in medical students after one-year enrollment of their training. This warrants further investigation.

We observed a high prevalence of depressive symptoms (20.7%) and suicidal ideation (14.7%). These estimates fall within the range of prevalence observed in previous studies among medical students, which equaled 14% to 31% in terms of depressive symptoms and 7% to 14% for suicidal thoughts [19,37,38,39]. The different estimated prevalence rates could be a result of the different instruments used in other studies, the different study time points of the medical students during their medical training, or actual difference between the medical students. Another possible explanation could be that students are less selected for mental health in their first year.

What makes the findings of this study particularly unique is the relationship of work-related personality Stability to depression and suicidal ideation. We found that scoring high on the work-related personality Stability factor may be a protective factor against the development of depressive symptoms and suicidal ideation, also when controlled for important covariates. Medical students who scored high on Stability had lower risk of depressive symptoms and reduced suicidal ideation. Although it remains unclear whether personality traits are cause or consequence, our findings are largely in line with previous studies that examined personality traits based on the Big-Five personality domain and NEO Personality Inventory Revised among samples of university students [19,40,41]. The inverse association of medical students scoring high on the Stability factor and better mental health may be explained by the fact that these individuals are more intrinsically resilient. Resilient individuals have the ability to deal robustly and calmly with academic and occupational pressures such as failures, excessive demands and stress [42]. Another possible explanation is that people exhibiting higher levels of Stability have learned to develop a resilient mindset and attitude. In other words, they have learned to withstand, adapt to, and recover from adversity and stress through training [43]. They have a largely positive outlook on life and only rarely feel weighed down by worries and fears. This latter notion was supported by the observation that university students, as well as individuals who scored higher on neuroticism, are at an increased risk for developing depression [21,22,44]. The detected association may also be related to other factors such as stressful life events [45]. Kendler et al. [45] reported that those with higher neuroticism were more likely to experience depression following stressful life events than those with lower neuroticism. Since our study is based on cross-sectional data, it is difficult to conclude whether depressive symptoms and suicidal ideation are a consequence of lower scores on the Stability personality trait. However, the fact that our findings indicate a higher score on the Stability personality factor, but not other work-related personality factors, is associated with lower risk of depressive symptoms and suicidal ideation point towards Stability as a protective factor.

This study also found that medical students with high scores on the Dominance factor are at greater risk for developing depression. This may be explained by social strains as people with high levels of dominance generally prioritize their own interests over those of others. In addition, they may have a greater number of interpersonal problems given that those with the Dominance personality trait can be argumentative and confrontational during a conflict situation [46].

Given that most lifetime mental disorders have their first onset during or shortly before the typical college age [47], the high prevalence of depression and suicidal ideation in this study highlighted the need to address mental health in medical students, particularly among those who score low on Stability and high on Dominance personality factors. Further, early successful treatment of mental disorders can also help decrease the risk for later dependence as mental disorders have been shown to predict later alcohol and other drug disorders [48,49]. Medical students are particularly at-risk for mental disorder since university years are also a period in which this group of emerging adults is exploring and forming their identity by trying out various life possibilities [50,51]. Early interventions may help to enhance the mental health of all medical students at different stages of their medical training [52,53,54]. For instance, it has been found that medical students who attended mind-body skills training program/cognitive behavioral therapy (CBT) based stress management interventions reported a significant reduction in stress [53,54]. Similarly, Krasner et al. [52] concluded that training medical students in mindfulness resulted in changes in their emotional stability.

Moreover, by reducing the risk of depression, the risk of low academic functioning and dropping out of university will also decrease [55]. Research is needed on identifying suitable stress management and coping strategies as medical students face not only the demands of their medical training, but also exposure to distressing clinical situations in their clinical placement, as well as personal stressors such as financial difficulties and moving away from family and friends often for the first time. Longitudinal research is also needed to determine the effectiveness of providing stress reduction training and resources for the prevention of common mental disorders in medical students.

Several limitations of this study should be mentioned. First, participants were from a single medical undergraduate training program and thus generalizability is uncertain. Second, responses were based only on self-reports of symptoms. Nonetheless, a clinically validated instrument was used. Third, this was a pooled cross-sectional study. As noted above, causal relationships between variables could not be confirmed as we were unable to account for omitted explanatory variables in cross-sectional analysis. Longitudinal research is recommended to verify the causal relationship between work-related personality factors with depressive symptoms and suicidal ideation. Fourth, the low response rate as well as nonresponse rates may limit the generalizability and representativeness of the sample. For example, some participants may have refused to take part in the study by not completing the survey or may have underreported their mental health problems. It is difficult to know for a fact what the most relevant reasons may be. However, based on our expertise and understanding of what we know about who participates in health research studies, we can speculate that medical students may not have taken part because they were not interested, found the research topic irrelevant, or had competing demands on their time. Although the rate of nonresponse may pose potential bias, it has been argued that there is no necessary relationship between response rate and degree of bias [56]. The lower response rate in our study is unlikely to have a large nonresponse bias because the characteristics of the responders and non-responders (e.g., most were of a similar age, socio-economic background and academic achievement) in our study do not differ significantly. Nonresponse bias also does not vary closely with nonresponse rates [56].

Despite these limitations, the findings provide insight into the prevalence rates of depression and suicidal ideation among medical students after one-year enrollment. In addition, the present study provides an explorative starting point for the generation of hypotheses on the relationship between personality traits in a professional context and mental health. To further validate the findings, another study with a larger random non-medical sample is recommended.

## 5. Conclusions

Symptoms of depression and suicidal ideation are prevalent in medical students after one-year of their medical training. Personality traits can provide insight into when increased attention—or support—is needed. Medical students with low Stability personality trait scores and those with high Dominance personality trait scores experience greater mental distress. Our results underline the important role of promoting mental health of medical students at early stages of their training, in particular to those who show higher prevalence rates of depression and score low on work-related Stability. Our research also highlights the need for effective interventions, e.g., CBT based stress management interventions—in conjunction with learning environment interventions. Medical Schools should reach out to their students by offering support to help them find the best coping strategies and ensuring a range of proper mental health services are in place. In addition, medical educators could also take environmental action by developing a better medical curriculum that addresses the various needs of the trainees. Further studies are needed to confirm these results and to evaluate the effectiveness of interventions.

## Figures and Tables

**Table 1:** Description of the focus of each of the 6 factors and facets as well as each factor’s ‘key’ question.

**Commitment** Facets: Career orientation, achievement motivation, competitiveness	How committed you are at pursuing professional goals?
**Discipline** Facets: Planning and organization, attention to detail, analytical approach	How carefully do you plan and work?
**Dominance** Facets: Potential, independence, readiness for conflict	How determined are you to pursue your own interests?
**Stability** Facets: Composure, self-confidence, tolerance to frustration	How well can you cope under pressure?
**Cooperation** Facets: Team orientation, willingness to compromise, ability to integrate	To what extent do you prefer to work as part of a team?
**Social competence** Facets: Sociability, Empathy, Enthusiasm	To what extent do you take an active role in social situation?

**Table 2:** Description of measures and sample (*n* max = 251).

Variable	Categories or Potential Range	%	Mean (SD)
Gender	Male	28.7	-
	Female	71.3	-
Age		-	22.22 (4.72)
Commitment ^a^	1–6	-	4.0 (0.67)
Discipline ^a^	1–6	-	4.37 (0.72)
Social competence ^a^	1–6	-	3.87(0.87)
Cooperation ^a^	1–6	-	3.35 (0.90)
Dominance ^a^	1–6	-	3.44 (0.80)
Stability ^a^	1–6	-	3.33 (0.81)
Partnership	Yes	51.4	-
	No	48.6	-
Financial status of Parents’ home	1–10	-	6.38 (2.18)
Depressive symptoms ^b^	Yes	20.7	-
	No	79.3	-
Suicidal ideation ^c^	Yes	14.7	-
	No	85.3	-

^a^ Work-related personality dimensions assessed by the BIP-6F. ^b^ Depressive symptoms assessed by PHQ-8. ^c^ Suicidal ideation assessed by PHQ-9 item 9.

**Table 3:** Associations of work-related personality dimensions, depression and suicidal ideation.

**Depressive Symptoms** ^a^	**Model 1**	**Model 2**	**Model 3**	
	**OR (95% CI)**	**OR (95% CI)**	**OR (95% CI)**	***n* = 251**
Commitment: High	0.81 (0.41–1.62)	0.89 (0.43–1.82)	0.86 (0.41–1.79)	
Discipline: High	0.56 (0.28–1.11)	0.59 (0.29–1.20)	0.58 (0.28–1.21)	
Social competent: High	0.71 (0.37–1.37)	0.63 (0.31–1.25)	0.75 (0.36–1.54)	
Cooperation: High	1.03 (0.53–1.98)	0.96 (0.48–1.91)	0.90 (0.45–1.81)	
Dominance: High	2.45 (1.27–4.73) **	2.57 (1.29–5.13) **	2.46 (1.22–4.97) **	
Stability: High	0.16 (0.08–0.34) ***	0.20 (0.09–0.43) ***	0.19 (0.09–0.42) ***	
Pseudo-R^2^ (Nagelkerke)	0.24	0.27	0.28	
**Suicidal Ideation** ^b^	**Model 4**	**Model 5**	**Model 6**	
	**OR (95% CI)**	**OR (95% CI)**	**OR(95% CI)**	***n* = 251**
Commitment: High	1.03 (0.47–2.26)	1.10 (0.50–2.46)	1.11 (0.49–2.48)	
Discipline: High	0.90 (0.41–1.96)	0.96 (0.44–2.13)	0.96 (0.43–2.14)	
Social competent: High	0.74 (0.35–1.57)	0.71 (0.33–1.51)	0.71 (0.32–1.53)	
Cooperation: High	0.51 (0.23–1.09)	0.49 (0.22–1.07)	0.49 (0.22–1.07)	
Dominance: High	2.05 (0.96–4.34)	2.05 (0.96–4.37)	2.03 (0.95–4.35)	
Stability: High	0.34 (0.15–0.75) **	0.38 (0.16–0.87) *	0.38 (0.16–0.87) *	
Pseudo-R^2^ (Nagelkerke)	0.10	0.14	0.14	

Model 1: unadjusted. Model 2: adjusted for age and gender. Model 3: adjusted for socio-demographic variables (partnership and status of parents’ home). * *p* < 0.05; ** *p* < 0.01; *** *p* < 0.001. Abbreviations: OR, Odds Ration; CI, Confidence Intervals. ^a^ Depressive symptoms assessed by PHQ-8. ^b^ Suicidal ideation assessed by PHQ-9 item 9.
